# Depression, Anxiety, Stress Symptoms and Health-Related Quality of Life in Hemodialysis Patients: Cross-Sectional Findings from a Romanian Cohort

**DOI:** 10.3390/medicina62020242

**Published:** 2026-01-23

**Authors:** Adriana-Luciana Luca, Felicia Militaru, Cristina Mariana Văduva, Ilie-Robert Dinu, Daniela Teodora Maria, Mădălina Iuliana Mușat, Virginia Maria Rădulescu, Ion Udriștoiu, Eugen Moța

**Affiliations:** 1 U.M.F. Doctoral School Craiova, University of Medicine and Pharmacy of Craiova, 200349 Craiova, Romania; eugenmota@yahoo.com; 2 Department of Psychiatry, University of Medicine and Pharmacy of Craiova, 200349 Craiova, Romania; felicia.militaru@umfcv.ro (F.M.); ion.udristoiu@umfcv.ro (I.U.); 3 Hemodialysis Center, Emergency County Hospital of Craiova, 200642 Craiova, Romania; cristina_vaduva2002@yahoo.com; 4 Department of Nephrology, University of Medicine and Pharmacy of Craiova, 200349 Craiova, Romania; robert.dinu@umfcv.ro (I.-R.D.); daniela.maria@umfcv.ro (D.T.M.); 5 Experimental Research Centre for Normal and Pathological Aging, University of Medicine and Pharmacy of Craiova, 200349 Craiova, Romania; madalina.musat@umfcv.ro; 6 Department of Scientific Research Methodology, University of Medicine and Pharmacy of Craiova, 200349 Craiova, Romania; 7 Department of Medical Informatics and Biostatistics, University of Medicine and Pharmacy of Craiova, 200349 Craiova, Romania

**Keywords:** chronic kidney disease, hemodialysis, depression, anxiety, stress, health-related quality of life, comorbidity

## Abstract

*Background and Objectives*: Chronic kidney disease (CKD) and maintenance hemodialysis (HD) are frequently associated with psychological distress and impaired health-related quality of life (HRQoL). However, the relationships between depressive, anxiety, and stress symptoms, clinical factors, and HRQoL remain insufficiently understood in routine care. This study aimed to assess the prevalence of psychological distress and to explore cross-sectional correlates of kidney disease-specific and generic HRQoL in Romanian patients receiving long-term HD, providing one of the first detailed characterizations of these relationships in an Eastern European maintenance HD cohort. *Materials and Methods*: This single-center cross-sectional study included 125 adult patients undergoing maintenance HD for at least one year. Baseline assessment comprised socioeconomic, demographic and clinical and paraclinical data, including Charlson Comorbidity Index (CCI), dialysis adequacy (spKt/V), cognitive function, psychological distress assessed with the Depression, Anxiety and Stress Scale (DASS-21R), and HRQoL evaluated using the Kidney Disease Quality of Life Short Form (KDQOL-SF™ 1.3). HRQoL domains and physical and mental component summary scores (PCS, MCS) were analyzed using descriptive statistics, correlation analyses, and multivariable linear regression. Follow-up assessments at approximately one year were summarized descriptively. *Results*: Disease-specific HRQoL revealed marked impairment in perceived disease burden and work status, while physical HRQoL was substantially reduced (PCS 36.5 ± 9.6). Mental HRQoL was relatively preserved (MCS 48.8 ± 8.8). At baseline, 48.0% of patients reported at least mild depressive symptoms, 34.4% anxiety symptoms, and 44.0% stress symptoms. spKt/V showed a modest association with PCS. Psychological distress demonstrated weak associations with HRQoL; stress was independently associated with lower MCS, with limited explained variance (R^2^ ≤ 0.15). *Conclusions*: Psychological distress is common among Romanian HD patients and is cross-sectionally associated with markedly impaired physical HRQoL. While the present design does not allow causal inferences, these findings support the implementation of routine psychological screening and the consideration of targeted psychosocial interventions in HD care.

## 1. Introduction

Chronic kidney disease (CKD) is defined by persistent (≥3 months) abnormalities in kidney structure and/or function, with severity determined based on the estimated glomerular filtration rate (eGFR). According to the Kidney Disease: Improving Global Outcomes (KDIGO) guidelines, stage 5 CKD corresponds to an eGFR < 15 mL/min/1.73 m^2^, indicating the need for renal replacement therapy (RRT)—either kidney transplantation, peritoneal dialysis (PD), or hemodialysis (HD) [1].

CKD represents a major public health issue associated with high costs due to its increasing incidence, elevated morbidity and mortality, and profound impact on patients’ quality of life (QoL). Worldwide, CKD affects more than 850 million people [2] and is projected to become the fifth leading cause of death by 2040 [3].

HD, essential for patient survival, entails numerous adverse effects and permanent lifestyle modifications that significantly correlates with QoL across physical, social, and mental health dimensions.

QoL is a multidimensional construct in the context of chronic disease, encompassing physical aspects (fatigue, sleep disorders, somatic symptoms such as pruritus, and hygienic–dietary restrictions), social aspects (tendency toward social isolation), functional limitations (disability, unemployment), existential and spiritual dimensions (coping with disease and redefining personal values), as well as mental health aspects (anxiety, depression, chronic stress, cognitive impairment) and treatment satisfaction. Together, these dimensions reflect the overall health-related quality of life (HRQoL) impact experienced by CKD patients [4].

Numerous studies have shown that the main variables significantly correlated with HRQoL scores include demographic factors (age, sex), clinical parameters (dialysis duration and frequency, comorbidities), socioeconomic status (SES), social and family support, and mental health [5,6,7,8]. Empirical evidence highlights a direct correlation between SES and HRQoL, as well as between SES and depression [9,10,11,12].

Recent literature has emphasized the need to implement valid and standardized instruments for assessing HRQoL among dialysis patients [13,14]. A systematic review identified the most frequently used instruments as the Kidney Disease Quality of Life Short Form, version 1.3 (KDQOL-SF™ 1.3), the World Health Organization Quality of Life—Brief Version (WHOQOL-BREF), and the Short Form-36 Health Survey (SF-36), which primarily assess physical, psychological, and social dimensions [15].

Studies have consistently demonstrated that HD patients score significantly lower in both the physical and psychological domains of HRQoL, which is associated with higher rates of hospitalization and mortality [6,16,17,18]. These marked decreases underscore the importance of psychosocial interventions.

The psychological component is notably impaired in CKD patients compared with individuals suffering from other chronic conditions [19]. High prevalence rates of depression and anxiety—often underdiagnosed—have been reported, both being among the key determinants of HRQoL and therapeutic adherence [20].

A 2024 meta-analysis reported a depression rate of approximately 30% among HD patients, higher than in PD patients, with variations depending on the assessment tools used [21].

When anxiety coexists with depression, treatment adherence declines [22]. Anxiety, identified by the World Health Organization (WHO) as the most common psychiatric condition, affected 359 million individuals globally in 2021, predominantly women [23]. Cross-sectional and prospective observational studies have shown that anxiety is correlated with lower HRQoL, frequent hospitalizations, and increased mortality [24,25,26]. A 2021 meta-analysis found that 19% of CKD patients exhibit symptoms of an anxiety disorder, while 40% experience clinical anxiety [27].

Depression profoundly alters HRQoL through its psychological, physical, and social components, manifesting as low mood, anhedonia, and apathy–abulia, whereas anxiety primarily impairs adaptation to disease and treatment and alters life perception.

Furthermore, HD patients experience significant chronic stress due to the interaction of biological, psychological, and social factors.

While depression and anxiety are inversely correlated with HRQoL, stress presents a bidirectional relationship: chronic stress negatively correlates with physical, psychological, social, and environmental dimensions [28], particularly adaptation capacity, motivation, and health perception, leading to sleep disturbances, multiple somatizations, and fatigue [29].

Stress in HD patients severely impairs the physical, psychological, and social dimensions of HRQoL (r = −0.53, *p* < 0.001) [26], primarily due to uncertainty about the future, dependence on dialysis, and disruption of daily routines [30], ultimately worsening general health and prognosis [29].

In addition to psychological impairment—which may represent both a consequence of dialysis and a comorbidity—HD patients often present with multiple systemic comorbidities that further reduce HRQoL [31]. A multicenter study showed that each one-point increase in the Charlson Comorbidity Index (CCI) results in a 2.5-point decrease in the physical HRQoL score [32].

All these variables are negatively associated with the HRQoL of HD patients and are interrelated with psychological well-being. Reliable and validated tools such as the Depression Anxiety and Stress Scale (DASS-21R) for assessing the triad most frequently present in HD patients, the KDQOL-SF™ 1.3 for kidney disease-specific HRQoL evaluation, and the Socioeconomic Scale (SES-3) for determining SES, along with correlations with the CCI, support the objective of our study: to quantify the burden of psychological symptoms and to explore the relationships between mental health, SES, comorbidity, and HRQoL in HD patients, in order to establish future therapeutic approaches emphasizing the need for multidisciplinary teams.

Against this background, data from Eastern European HD populations, and particularly from Romania, remain scarce, especially regarding studies that jointly examine psychological symptoms, comorbidity burden, dialysis adequacy (spKt/V), SES, and kidney disease-specific HRQoL. The present study therefore aimed to (i) estimate the prevalence and severity of depression, anxiety, and stress symptoms in a cohort of long-term HD patients from a single Romanian center using the DASS-21R, and (ii) explore the cross-sectional associations between these psychological dimensions and KDQOL-SF™ 1.3 domains, after accounting for CCI, spKt/V, and SES. By integrating these domains in the same analytical framework, we sought to clarify to what extent psychological distress explains variability in physical and mental component summaries of HRQoL (PCS and MCS) in a relatively well-dialyzed survivor cohort.

## 2. Materials and Methods

### 2.1. Study Design and Participants

This cross-sectional study with two assessment time points was conducted over a two-year period (December 2021–November 2023), at the DIAVERUM Nephrology and Dialysis Center in Craiova, Romania. The target population consisted of adult patients with stage 5 CKD receiving maintenance HD three times per week for at least one year.

The initial study cohort consisted of 220 participants diagnosed with stage 5 CKD who met the basic eligibility criteria for maintenance HD in the center. Patients were excluded if they met one or more of the following criteria: a Mini-Mental State Examination (MMSE) score ≤ 20, indicating relevant cognitive impairment (n = 12); a history of psychiatric disorders under psychotropic medication, including depressive disorder or schizophrenia (n = 7); congenital or acquired conditions that impaired comprehension or communication, such as moderate-to-severe intellectual disability or congenital deafness (n = 2); voluntary withdrawal from the study due to fatigue or the length of the assessment process (n = 54); or death during the study period (n = 20). After applying these criteria, 125 patients remained eligible and completed the initial assessment.

Since the present work is a cross-sectional analysis based on this clinical cohort, it was important to verify that the available sample size was adequate to detect clinically meaningful associations. The minimum required sample size was estimated using the software application G*Power 3.1.9.7 (Heinrich Heine University Düsseldorf, Germany) for a two-sided correlation test between psychological distress (DASS-21R domain scores) and HRQoL (PCS and MCS). Assuming a medium effect size (ρ = 0.30), a significance level α of 0.05, and a desired power (1 − β) of 0.90, the calculation yielded a minimum required sample size of 113 participants.

**Figure 1:**
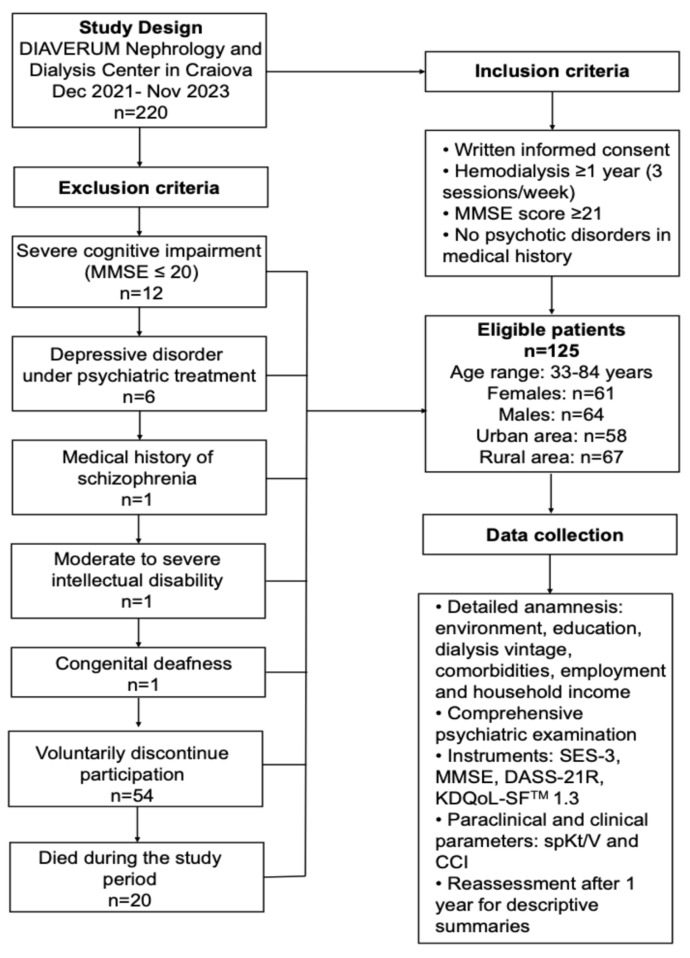
Study design and data collection process. Flowchart illustrating study period, inclusion and exclusion criteria, sample characteristics and data collection methods applied to 125 HD patients (61 females, 64 males; age range: 33–84 years) at the DIAVERUM Nephrology and Dialysis Center in Craiova (December 2021–November 2023) (created using Adobe InDesign 21.0.1).

For the present HRQoL-focused analysis, we further restricted the sample to patients with complete baseline KDQOL-SF™ 1.3 data. The final analytic cohort comprised 125 patients, of whom 61 (48.8%) were female and 64 (51.2%) were male; 58 (46.4%) lived in urban areas and 67 (53.6%) in rural areas. Ages in this analytic cohort ranged from 33 to 84 years. All analyses reported in this article refer to this baseline cross-sectional sample; scores obtained at the second assessment time point (approximately one year later) were used only for descriptive summaries (Figure 1).

### 2.2. Inclusion Criteria

Participants were included if they had end-stage CKD and had been receiving HD treatment for at least one year, with a dialysis frequency of three sessions per week, and demonstrated the ability to understand and respond to the questionnaires in Romanian. The one-year treatment threshold was considered sufficient for patients to have adapted to their new lifestyle and the dialysis process, as well as for the onset of the most common psychological disorders identified in this population of chronically ill patients.

### 2.3. Exclusion Criteria

Exclusion criteria included the following: MMSE score ≤ 20, indicative of moderate or severe neurocognitive impairment; a medical history of psychiatric disorders under psychotropic medication (as such treatment may improve mental health status and confound results); conditions impairing comprehension or communication; and voluntary withdrawal from the study.

### 2.4. Data Collection Procedure

Written informed consent was obtained from each participant prior to data collection. The data collection process consisted of three primary components:

(1) Clinical, socioeconomic, and demographic profile: data regarding sex, age, area of residence, medical history, HD duration, social and family support, and SES, assessed via the SES-3 scale were obtained through detailed anamnesis.
(2) Administration of standardized psychometric instruments in two phases (baseline and one-year follow-up): MMSE—to identify neurocognitive impairment that could compromise the study; DASS-21R—Romanian-adapted and standardized version, to assess the most frequent psychiatric conditions among HD patients; KDQOL-SF™ 1.3—to evaluate HRQoL.
(3) Clinical and paraclinical parameters provided by the DIAVERUM Nephrology and Dialysis Center: CCI; spKt/V.

### 2.5. Variables and Measurements

SES was assessed using the SES-3 scale, which includes three dimensions: (1) education, (2) occupation, and (3) household monthly income. Each domain was rated on a three-point ordinal scale (0–2), yielding a total SES score ranging from 0 to 6. Based on the cumulative score, participants were classified as having low (0–2), medium (3–4), or high (5–6) SES [33]. Educational level was scored as 0 for primary or secondary education, 1 for completion of high school, post-secondary, or vocational training, and 2 for university or postgraduate education. Occupational status was coded as 0 for unskilled labor, manual work, agricultural work, or unemployment; 1 for administrative, service, or sales-related positions; and 2 for professional, entrepreneurial, or managerial occupations. Household income was rated as 0 when below the national threshold (less than 50% of the national median income or below the minimum wage), 1 for income between 50% and 100% of the national median, and 2 for income exceeding the national median or more than twice the minimum wage.

Cognitive function was evaluated using the 11-item MMSE, developed by Marshall Folstein. This standardized and reliable instrument assesses multiple cognitive domains—orientation, memory, attention, language, and visual constructive abilities—yielding a total score between 0 and 30. Scores ≤ 20 indicate moderate to severe cognitive impairment.

To assess the main mental health dimensions observed in HD patients—depression, anxiety, and stress—DASS-21R was employed. The instrument comprises three distinct subscales, each evaluating one of the emotional dimensions, with 21 items rated on a 0–3 Likert scale [34]. The depression subscale measures dysphoria, hopelessness, anhedonia, lack of motivation and energy, devaluation of life, and low self-esteem. The anxiety subscale evaluates autonomic arousal, musculoskeletal symptoms, situational anxiety, and subjective anxiety perception. The stress subscale captures chronic nonspecific arousal, measuring psychological tension, irritability, and hyperactivity. For each DASS-21R subscale, raw scores ranging from 0 to 21 were calculated. Subsequently, individual raw scores were transformed into sex- and age-adjusted z-scores, in accordance with the guidelines provided in the DASS-21R manual. These standardized scores were used as continuous predictors in the correlation and regression analyses. Symptom severity was categorized based on z-score thresholds as follows: normal (≤0.5), mild (>0.5 to <1.0), moderate (≥1.0 to <2.0), severe (≥2.0 to <3.0), and extremely severe (≥3.0). Z-score standardization reduces distributional bias and facilitates a more accurate evaluation of symptom severity. The DASS-21R has been validated for the Romanian population, ensuring both cultural and psychometric relevance, and data collection followed the standardized manual guidelines [35].

HRQoL was assessed using the KDQOL-SF™ 1.3, a valid and reliable instrument developed by Hays in 1997 at the RAND Corporation (Santa Monica, CA, USA), validated and translated into Romanian by the Romanian Society of Nephrology. This tool is specifically designed for patients undergoing RRT and combines general health evaluation with kidney disease-specific domains. The KDQOL-SF™ 1.3 includes the 36 items of the SF-36—addressing general health, physical functioning, role limitations, pain, vitality, social functioning, and mental health—plus 43 additional items targeting kidney-specific aspects such as symptom burden, treatment effects, perceived disease burden, social support, quality of interaction with medical staff, sleep, and satisfaction with care. Responses to KDQOL-SF™ 1.3 were scored following established scoring principles. Raw item responses, recorded using a combination of dichotomous formats and Likert-type scales with three, five, or six response options, were first examined for directionality and, where necessary, reverse-coded so that higher values consistently reflected better HRQoL. Subsequently, all item responses were linearly transformed to a standardized 0–100 scale based on the range of possible responses for each item [36]. Domain scores were calculated by averaging the transformed item scores within each corresponding scale, provided that at least half of the items in that domain were completed. Kidney disease-specific domains and generic health domains were analyzed separately, and no overall summary score for the entire instrument was computed. PCS and MCS were derived using the SF-12 scoring algorithm applied to the SF-36 core embedded within the KDQOL-SF™ 1.3.

To estimate long-term mortality risk and explore correlations among patients, the CCI—developed by Dr. Mary Charlson and colleagues in 1987—was used [37]. This composite clinical index evaluates 19 categories of chronic diseases, assigning a weight to each based on severity and impact on survival. The total CCI score is obtained by summing individual scores: 0 (no comorbidities); 1–2 (low comorbidity); 3–4 (moderate comorbidity); and ≥5 (high comorbidity, high mortality risk).

Dialysis efficacy was assessed using the single-pool Kt/V (spKt/V), a key paraclinical parameter of HD adequacy. It measures how many times, during one dialysis session, the total body urea distribution volume has been cleared. The calculation is based on three components: blood urea clearance (K), dialysis session duration (t), and the urea distribution volume (V)—approximately 55–60% of body weight, corresponding to total body water [38,39]. Interpretation thresholds were as follows: spKt/V < 1.2—inadequate dialysis with increased risk of complications and mortality; spKt/V between 1.2 and 1.4—adequate dialysis; spKt/V > 1.4—optimal level. Values > 1.6–1.8 do not provide additional clinical benefits and may increase the risk of hypotension, protein catabolism, and inefficient resource utilization [40].

CCI and spKt/V data were provided by the DIAVERUM Nephrology and Dialysis Center, with institutional approval.

### 2.6. Statistical Analysis

Statistical analyses were performed using IBM SPSS Statistics, version 26 (IBM Corp., Armonk, NY, USA). Data were first checked for accuracy, missing values and outliers. All inferential analyses were conducted on complete-case data; no imputation of missing values was undertaken. Continuous variables were summarized as mean ± standard deviation (SD), median and/or interquartile range [IQR], and range, as appropriate. Categorical variables were presented as absolute frequencies and percentages.

The primary analyses focused on the baseline assessment. For the DASS-21R, scores were treated both as continuous domain scores and as severity categories (normal, mild, moderate, severe, extremely severe) according to the scoring manual. At baseline, the prevalence of each severity category was described overall, and χ^2^ tests of independence were used to compare the full five-level distributions of depression, anxiety and stress across sex and age classes. For continuous analyses, the age- and sex-adjusted z-scores described in Section 2.5 were used.

HRQoL was analyzed using the KDQOL-SF™ 1.3 instrument, with all domain scores expressed on a 0–100 scale, where higher values indicate better perceived health. Kidney disease-specific and generic domains were described using the same summary statistics as above. The PCS and MCS were considered the main HRQoL outcomes. Differences in PCS and MCS across categories of dialysis adequacy (spKt/V < 1.2, 1.2–1.4, >1.4) were examined using Kruskal–Wallis tests, given the non-normal distribution of composite scores.

Bivariate associations between DASS-21R domain scores and selected HRQoL domains (burden of kidney disease, global health ratings, general health, energy/fatigue, PCS, MCS, role-emotional), as well as clinical and socioeconomic variables (SES-3 score, CCI, spKt/V, age), were assessed using Spearman’s rank correlation coefficients (ρ), with corresponding two-sided *p*-values.

To identify independent correlates of HRQoL, two multivariable linear regression models were fitted with PCS and MCS at the baseline assessment as dependent variables. Covariates were entered simultaneously and included age class (<50, 50–64, ≥65 years, with <50 years as the reference category), sex, SES score, CCI, spKt/V, and the three DASS-21R domain scores. Because SES-3 already incorporates education, occupation, and household income, the SES-3 total score was used in the primary HRQoL models and the SES components were not entered simultaneously to avoid collinearity. In addition, to explore independent correlates of psychological distress, we fitted multivariable proportional-odds ordinal logistic regression models using the five-level DASS-21R severity categories (normal, mild, moderate, severe, extremely severe) as outcomes. In these models, DASS-21R depression, anxiety and stress were entered as standardized z-scores, so that β coefficients reflect the change in PCS/MCS per 1-SD increase in each domain. As exploratory sensitivity analyses, we also fitted alternative models in which each DASS-21R domain was entered separately, as well as a model using a composite psychological distress index in place of the three subscales, to assess the robustness of the associations between anxiety, stress and HRQoL. Results are reported as unstandardized regression coefficients (β) with 95% confidence intervals (CI), *p*-values and coefficients of determination (R^2^). Model assumptions (linearity, homoscedasticity, approximate normality of residuals and absence of problematic multicollinearity) were checked using residual plots and variance inflation factors. The distributions of PCS and MCS were inspected and showed deviations from normality. Therefore, for linear regression we evaluated the normality assumption primarily at the level of model residuals (Q–Q plots), which indicated approximate residual normality adequate for inference. For these ordinal models, sex (female vs. male), place of residence (urban vs. rural), age (years), dialysis vintage (years), education score, occupational score, income score, CCI, spKt/V, and MMSE were entered simultaneously. Effects are reported as adjusted odds ratios (aOR) with 95% confidence intervals and two-sided *p*-values. The proportional-odds (parallel lines) assumption was evaluated, and model diagnostics were examined to ensure the adequacy of inference.

Scores obtained at the one-year re-evaluation were summarized descriptively using the same statistics as for baseline. No formal tests of within-patient change between the two time points were conducted, and one-year data were not used in multivariable models. With an analytic sample of 125 patients, the study had approximately 90% power at α = 0.05 to detect medium-sized correlations (ρ ≈ 0.30) between DASS-21R scores and HRQoL outcomes; smaller effect sizes should therefore be interpreted with caution. All statistical tests were two-sided, and *p* < 0.05 was considered statistically significant. Given the exploratory nature of the study and the relatively large number of correlations and regression coefficients examined, no formal adjustment was made for multiple comparisons. Therefore, individual *p*-values should be interpreted descriptively, with emphasis placed on the overall pattern and magnitude of effects rather than on any single statistically significant result.

### 2.7. Research Ethics

The study was conducted in accordance with the Declaration of Helsinki and was approved by the Ethics Committee of the University of Medicine and Pharmacy of Craiova, Romania (Approval No. 177/29.10.2021). All participants provided written informed consent prior to enrollment. In cases where severe depression, anxiety, or stress were identified during the study, requiring immediate intervention, treatment was offered in accordance with the current National Institute for Health and Care Excellence (NICE) guidelines [41,42,43].

## 3. Results

### 3.1. Sociodemographic and Clinical Characteristics

A total of 125 HD patients were included in the cross-sectional analysis. Overall, the cohort was composed predominantly of older adults (mean age 60.78 ± 11.90 years; median 63.00 [IQR 54.00–69.00], range 33.00–84.00) with substantial exposure to long-term dialysis treatment (dialysis vintage 6.00 ± 4.63 years; median 5.00 [2.00–9.00], range 1.00–20.00). Women and men were almost equally represented (48.80% vs. 51.20%). Slightly more than half of participants lived in rural areas (53.60%), and educational attainment was largely low to moderate, with only a minority reporting university education (14.40%). Socioeconomically, the sample clustered mainly in the medium range, consistent with a mean SES score of 3.14 ± 1.31 on the 0–6 scale (median 3.00 [2.00–4.00], range 1.00–6.00).

Clinically, patients exhibited a high comorbidity burden, with a CCI of 5.42 ± 2.10 (median 5.00 [4.00–7.00], range 2.00–11.00); categorically, 64.80% were classified in the CCI ≥ 5 group, while 26.40% were in the 3–4 category and 8.80% in the 1–2 category. Dialysis adequacy was generally favorable: mean spKt/V was 1.60 ± 0.34 (median 1.59 [1.36–1.86], range 0.85–2.32), with 71.20% of patients having spKt/V > 1.4, 16.80% in the borderline range (1.2–1.4), and 12.00% below 1.2. Global cognitive function was preserved, consistent with the predefined exclusion criteria, as indicated by MMSE values tightly clustered at the upper end of the scale (mean 27.36 ± 1.94; median 28.00 [25.00–29.00], range 25.00–30.00).

Sociodemographic and clinical characteristics can be observed in Table 1. Taken together, these baseline data describe an older, highly comorbid HD population, living predominantly in rural or semi-rural settings, with modest educational and socioeconomic resources but largely adequate technical dialysis parameters.

### 3.2. Prevalence of Depression, Anxiety, and Stress Symptoms

The psychological profile of the cohort indicated a substantial burden of mood symptoms. On the standardized DASS-21R scale, mean scores for depression, anxiety and stress were 0.92 ± 1.78, 0.26 ± 1.17 and 0.45 ± 1.48, respectively, with medians close to zero for all three domains (depression: 0.41 [IQR −0.63–2.28]; anxiety: −0.08 [−0.68–0.91]; stress: 0.00 [−0.78–1.43]). This pattern suggests that many patients reported few or no symptoms, while a non-negligible subgroup experienced clinically relevant distress, resulting in a wide spread of values across the sample.

**Figure 2:**
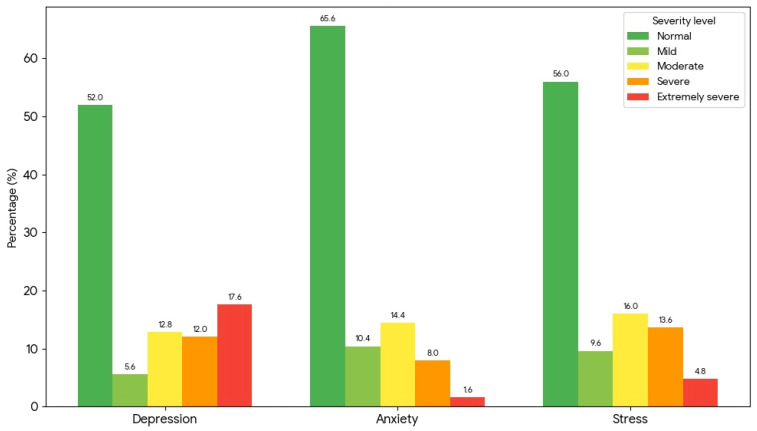
Overall, 48.0% of patients reported at least mild depressive symptoms, 34.4% at least mild anxiety, and 44.0% at least mild stress. Clinically relevant symptomatology was also frequent: approximately 42% of the cohort fell in the moderate-to-extremely severe range for depression, 24% for anxiety, and 34% for stress. Thus, even though many individuals scored within the normal range, a substantial subgroup experienced marked psychological distress, underscoring the need for systematic psychological assessment in routine HD care.

Categorical DASS-21R severity scores highlighted a relatively high burden of symptoms in this cohort (Figure 2).

The psychological profile of the cohort indicated a substantial burden of mood-related symptoms, alongside marked heterogeneity. On the standardized DASS-21R scale, mean scores for depression, anxiety, and stress were 0.92 ± 1.78, 0.26 ± 1.17, and 0.45 ± 1.48, respectively, with medians close to zero across all three domains (depression: 0.41 [IQR −0.63–2.28]; anxiety: −0.08 [−0.68–0.91]; stress: 0.00 [−0.78–1.43]). This distribution suggests that many patients reported few or no symptoms, whereas a clinically relevant subgroup experienced substantial distress, generating a wide spread of values.

To preserve the clinical nuance of the five-category DASS-21R spectrum, we then explored the full severity distributions across sex and age classes without collapsing categories (Table 2). Depression severity showed only a non-significant trend toward association with sex (global χ^2^ *p* = 0.063) and no statistically significant association across age groups (*p* = 0.264), although the 50–64-year group displayed the highest proportion of extremely severe depression. In contrast, anxiety severity differed by both sex (χ^2^ *p* = 0.047) and age class (χ^2^ *p* = 0.022), with a shift toward more moderate-to-severe categories in men and in the 50–64-year group. For stress, severity patterns were broadly comparable by sex (χ^2^ *p* = 0.498) and across age classes (*p* = 0.518). Taken together, these findings suggest that, within this HD cohort, anxiety symptoms exhibit clearer stratification by sex and age than depressive or stress symptoms when the full DASS-21R severity spectrum is considered.

### 3.3. HRQoL (KDQOL-SF™ 1.3)

Among kidney disease-specific scales, the highest mean scores were observed for social support, cognitive function, dialysis staff encouragement and the symptoms/problems list (Table 3). These values suggest that, from the patient’s perspective, symptom management, perceived support from the dialysis team and basic cognitive functioning are relatively well preserved.

In contrast, burden of kidney disease scored substantially lower, pointing to a strong subjective sense that kidney disease interferes with daily life. The most impaired domain was work status, reflecting the high proportion of patients who are no longer in active employment or report severe limitations in their ability to work. These patterns mirror the clinical profile of an older, comorbid HD population, where daily activities and professional roles are profoundly altered even when support and relationships are perceived as good.

Physical functioning and role-physical were clearly impaired, in line with the high comorbidity burden and long dialysis vintage. The SF-36 general health scale underscored a globally reduced perception of physical health. By contrast, emotional well-being, role-emotional and social functioning showed more favorable values, consistent with only partial overlap between mood symptoms and perceived psychosocial functioning.

The composite scores reinforced this pattern. The PCS was clearly below the reference of 50 and indicating marked physical impairment at the cohort level. The MCS was comparatively higher, suggesting that while mental health is not fully preserved, it is less compromised than physical health.

To place these global scores in a clinical context, we examined PCS and MCS across categories of spKt/V, as measured by spKt/V. Patients with inadequate dialysis (spKt/V < 1.2) had the lowest PCS (30.35 ± 7.94), whereas those with borderline adequacy (1.2–1.4) showed the highest PCS (38.95 ± 9.91). Patients with spKt/V > 1.4 had intermediate PCS values (36.85 ± 9.47). The overall difference in PCS across spKt/V categories was statistically significant (Kruskal–Wallis *p* = 0.014), suggesting that better spKt/V is linked to improved perceived physical health, although extremely high spKt/V values did not translate into a further gain in PCS.

In contrast, the MCS did not differ meaningfully across spKt/V categories (*p* = 0.634), with mean values clustering around 48–50 in all three groups (50.09 ± 7.73, 47.59 ± 8.17 and 48.93 ± 9.09 for <1.2, 1.2–1.4 and >1.4, respectively). Additional analyses indicated no statistically significant differences in PCS or MCS across CCI categories or SES levels (all *p* > 0.150). Thus, within this cohort, spKt/V emerges as the main clinical correlate of physical HRQoL, whereas mental HRQoL appears less sensitive to technical dialysis parameters and more strongly linked to mood symptoms themselves, as explored in subsequent sections.

### 3.4. Associations Between Mood Symptoms, HRQoL, and Clinical Parameters

To explore how psychological distress relates to perceived health and disease burden, we examined bivariate associations between DASS-21R depression, anxiety and stress scores, and selected KDQOL-SF™ 1.3 domains (burden of kidney disease, global health ratings, energy/fatigue, PCS and MCS), as well as SES, CCI, spKt/V and age. Spearman’s rank correlations (ρ) were used throughout. The corresponding correlation coefficients and *p*-values are summarized in Table 4.

Overall, correlations between depression scores and HRQoL domains were weak and did not reach statistical significance. This suggests that, in this cohort, cross-sectional variation in depressive symptom scores captures only a limited proportion of the variability in HRQoL.

In contrast, anxiety scores displayed consistently small but statistically significant correlations with several HRQoL indicators (Table 4). Higher anxiety scores were associated with modest shifts in KDQOL-SF^TM^ 1.3 burden of kidney disease, KDQOL global health rating, SF-36 general health and energy/fatigue. Given their small magnitude, these findings are best interpreted as weak statistical associations, indicating only limited overlap between anxiety and how patients appraise their overall health and vitality. By contrast, correlations between anxiety and the composite scores remained small and did not reach conventional statistical significance.

Stress scores showed a similarly weak pattern of associations. The largest correlation was observed with the KDQOL-SF^TM^ 1.3 role-emotional domain, suggesting that elevated stress is linked to some restriction in role functioning due to emotional problems.

Finally, none of the DASS-21R domain scores showed meaningful associations with key clinical and socioeconomic indicators. Correlations with SES score, CCI, spKt/V and age were uniformly small (all |ρ| ≤ 0.16, all *p* ≥ 0.069), indicating that, within this cross-sectional snapshot, psychological symptom severity is only weakly structured by these background factors.

Overall, depressive symptoms show little cross-sectional coupling with the HRQoL indicators and clinical parameters examined, a pattern that will be further explored in multivariable models.

### 3.5. Multivariable Predictors of Physical and Mental HRQoL

To identify independent correlates of HRQoL, two multivariable linear regression models were fitted with the PCS and MCS scores as dependent variables. Covariates included age class (<50 years, 50–64 years, ≥65 years), sex, SES score, CCI, spKt/V, and the three DASS-21R domain scores (depression, anxiety, and stress) (Table 5).

For PCS, the overall model explained a modest proportion of variance. There was a non-significant tendency toward lower PCS in older age classes compared with patients < 50 years. Among psychological variables, higher anxiety scores were associated with slightly higher PCS, whereas depression and stress scores were not independently related to PCS in this model (both *p* > 0.10). Given the low R^2^ and non-significant global test, these associations should be interpreted with caution and viewed as exploratory.

For MCS, the multivariable model accounted for a somewhat larger, though still modest, proportion of variance. Again, age class, sex, SES, CCI and spKt/V were not significantly associated with MCS after adjustment (all *p* ≥ 0.24). In contrast, psychological variables emerged as the main correlates: anxiety and stress scores showed independent associations with MCS in opposite directions. Higher anxiety scores were related to higher MCS, whereas higher stress scores were associated with lower MCS. Given the small magnitude of these coefficients, their emergence only in the presence of simultaneous adjustment for the other DASS-21R domains, and the known intercorrelations among depression, anxiety and stress scores, these positive associations for anxiety are likely influenced by collinearity and should not be interpreted as indicating a protective effect.

Overall, the multivariable analyses indicate that, within this cohort, sociodemographic and somatic disease indicators (age, CCI, spKt/V, SES) contribute only modestly to cross-sectional variation in HRQoL, while psychological distress—particularly stress, and to a lesser extent anxiety—shows small but independent associations with mental HRQoL. The effect sizes are limited and the models explain a relatively small proportion of the variance, underscoring the exploratory nature of these findings and the need for cautious interpretation.

### 3.6. Exploratory Multivariable Correlates of Psychological Distress

To address whether key sociodemographic and clinical factors independently relate to psychological distress, we fitted proportional-odds ordinal logistic regression models using the five-level DASS-21R severity categories as outcomes and entered sex, place of residence, age, dialysis vintage, education score, occupational score, income score, CCI, spKt/V, and MMSE simultaneously (Table 6). Higher depression severity was independently associated with female sex and higher occupational score, while higher education, higher income, and better cognitive performance were protective. Dialysis vintage showed a borderline association with depression severity. For anxiety severity, only the occupational score remained independently associated. No independent correlates were identified for stress severity (urban residence showed a non-significant trend).

### 3.7. Descriptive Overview at the One-Year Re-Evaluation

At approximately one year, all 125 patients from the original cohort were re-assessed using the same DASS-21R and KDQOL-SF™ 1.3 instruments. Mean (±SD) one-year DASS-21R scores were 1.11 ± 1.89 for depression (median 0.41 [IQR −0.47–2.56]), 0.35 ± 1.27 for anxiety (median 0.19 [−0.58–0.96]), and 0.50 ± 1.53 for stress (median 0.27 [−0.78–1.63]). Overall, the pattern of psychological distress appeared broadly similar to baseline, with only small descriptive shifts across severity categories.

The HRQoL profile at one year also remained largely consistent with the baseline distribution. Kidney disease-specific domains continued to be among the most modified, including burden of kidney disease (mean 50.60 ± 18.21; median 50.00 [37.50–62.50]) and work status (mean 3.60 ± 12.98; median 0.00 [0.00–0.00]). The KDQOL-SF™ 1.3 global health rating was 71.04 ± 11.13 (median 70.00 [60.00–80.00]). For the SF-36 core components, general health was 43.96 ± 14.90 (median 45.00 [35.00–50.00]) and energy/fatigue was 58.80 ± 12.14 (median 60.00 [55.00–65.00]). Consistent with baseline, physical functioning remained more compromised than mental well-being, as reflected by a lower PCS (39.14 ± 9.99; median 38.92 [31.78–47.11]) compared with the MCS (47.91 ± 6.64; median 49.20 [42.03–52.87]).

Taken together, these descriptive data suggest relative short-term stability of psychological distress and HRQoL patterns among patients assessed at one year. However, no formal within-patient tests of change were performed; consequently, these one-year findings should be interpreted as a secondary descriptive snapshot rather than as evidence of longitudinal trajectories.

## 4. Discussion

Our study shows a high prevalence of depression (48.00%), anxiety (34.40%), and stress (44.00%) among patients undergoing HD. These estimates likely represent a lower bound, as patients with marked cognitive impairment, major psychiatric disorders under active psychotropic treatment, and those who died during the study period were excluded from the analytic cohort. However, these results are consistent with the findings of studies from the last three years in the literature that used DASS-21 among HD patients, reporting approximate values between 27–75% for depression, 38–75% for anxiety, and 20–67% for stress [44,45,46,47,48]. Beyond confirming these high prevalence estimates, our analysis provides additional, context-specific information. First, it documents this burden of depression, anxiety, and stress in an Eastern European HD cohort with relatively high spKt/V and systematic measurement of SES, CCI and HRQoL. Second, by modelling DASS-21R subscales together with CCI, spKt/V and SES, we show that psychological distress explains only a modest proportion of PCS and MCS variance, and that the expected strong negative depression–HRQoL coupling is attenuated in this survivor cohort. Third, the partially divergent behavior of anxiety and stress in multivariable models suggests that different dimensions of distress may have non-identical relationships with HRQoL once overlapping variance is taken into account; this pattern is hypothesis-generating and warrants cautious interpretation.

In a multicentric study on a group of 1332 patients undergoing HD and PD, DASS-21 proved to be a useful and reliable instrument, recording higher values in HD patients compared to PD, with the stress dimension predominating (48%), followed by depression (37%) and anxiety (20%) [19].

Recent studies show that SES, especially education and income, act as protective factors for mental health and HRQoL, facilitating treatment adherence and the social dimension [49,50,51]. In the present study, most patients fell into the low (34.4%) or medium (52.0%) SES categories, and lower SES levels tended to be accompanied by poorer HRQoL scores, although correlations were small and did not reach statistical significance, which is directionally consistent with these reports.

In our study, the cross-sectional associations between DASS-21R scores and KDQOL-SF™ 1.3 domains were generally weak. As detailed in Section 3.4, correlations between depressive symptoms and most HRQoL domains were small and often non-significant, whereas anxiety and stress showed only modest links with general health, energy/fatigue and role-emotional functioning. These effect sizes are clearly smaller than those reported in several previous HD cohorts and meta-analyses, in which depression and anxiety have emerged as moderate-to-strong determinants of impaired HRQoL [19,21,32,51,52,53]. This discrepancy may partly reflect differences in case mix and study design: our sample consisted of prevalent, clinically relatively stable HD patients with high comorbidity but largely adequate spKt/V, and patients with marked cognitive impairment or recent psychiatric treatment were excluded, which may have led to an under-representation of the most fragile cases. In addition, we used age- and sex-standardized DASS-21R z-scores and KDQOL-SF™ 1.3 domain scores, whereas many earlier studies relied on raw HADS or BDI scores and generic SF-36 outcomes; differences in instruments, scoring and variance range can influence the strength of observed associations. Finally, several potentially important mediators of the mood–HRQoL link—such as inflammatory biomarkers, pain burden, coping strategies and social support—were not measured in our study; these unobserved variables may account for part of the HRQoL variance that is attributed to depression and anxiety in other datasets [11,19,25]. However, in multivariable models that included all three DASS-21R domains simultaneously, anxiety showed a weak positive association with PCS and MCS, whereas stress remained inversely related to MCS.

This counterintuitive pattern for anxiety should not be interpreted as evidence that higher anxiety improves HRQoL. The three DASS-21R subscales are known to be moderately correlated, and the small positive regression coefficients for anxiety emerged only after adjustment for depression and stress, in a context where bivariate correlations between anxiety and PCS/MCS were small and non-significant. Such a configuration is compatible with suppression and collinearity effects, whereby overlapping variance between highly related predictors can invert or attenuate individual coefficients without reflecting a true protective role. Consistent with this interpretation, exploratory sensitivity analyses in which each DASS-21R domain was entered separately, as well as a model using a composite psychological distress index, suggested that the anxiety–HRQoL association is sensitive to model specification. We therefore treat the positive coefficients for anxiety as hypothesis-generating and interpret them with particular caution.

Lower scores were recorded especially for the burden of kidney disease and work status components of the KDQOL-SF™ 1.3, with mean scores of 43.90 ± 18.13 and 5.60 ± 17.06, respectively. These markedly impaired domains underscore the extent to which kidney disease interferes with daily life and employment.

The use of the KDQOL-SF™ 1.3 questionnaire allowed a comprehensive evaluation of HRQoL in patients undergoing HD treatment, integrating both the general health components and the dimensions specific to kidney disease (symptoms, treatment burden, interaction with medical staff) [54].

Our results indicate that the burden-of-kidney-disease and work-status dimensions recorded the lowest HRQoL scores, with means of 43.90 ± 18.13 and 5.60 ± 17.06, respectively. This pattern reflects the cumulative correlates of advanced kidney pathology, chronic symptomatology and the rigid HD schedule on patients’ everyday lives. In the meta-analysis by Raoofi et al. [55], pooled KDQOL-SF™ scores in HD and PD populations were in the mid-60s (95% CI approximately 55–73), indicating a generally moderate level of HRQoL. Against this backdrop, the very low burden and work-status scores observed in our cohort suggest an even more pronounced disruption of daily functioning, whereas other KDQOL-SF™ domains in our sample (symptoms, social support, cognitive function) fall closer to the ranges reported in that review.

In our cohort, the physical component of HRQoL were clearly impaired, with an PCS of 36.49 ± 9.61 and KDQOL-SF™ physical functioning and work-status scores of 58.36 ± 23.75 and 5.60 ± 17.06, respectively. These values suggest major limitations in physical activity and a considerable association of the disease on daily functioning [56]. In the study by Fructuoso et al. [57], conducted on 100 patients with CKD on HD, the mean score for “Work Status” was 30.00 ± 33.33 and for “Physical Functioning” 45.30 ± 12.39, also indicating reduced physical performance compared to the general population. Taken together, these findings show that physical HRQoL is consistently and substantially compromised in HD populations, with our cohort displaying particularly low work-status scores alongside markedly reduced, although somewhat variable, physical functioning, similar to recent literature [58].

In our study, the social dimension of HRQoL showed a moderate degree of impairment. SF-36 social functioning scored 64.60 ± 23.59, while the KDQOL-SF™ quality-of-social-interaction and social-support scales had means of 80.75 ± 13.79 and 88.53 ± 21.52, respectively. Although basic interactions and perceived support remain relatively preserved, these scores indicate that the HD regimen substantially constrains broader social participation, in line with recent reports describing secondary reductions in the psychosocial component of HRQoL [6,56]. The strict dialysis schedule, dietary restrictions and feelings of stigmatization are likely to contribute to reduced perceived social support and to the narrowing of patients’ social networks.

The relationship between the comorbidity burden, as followed in our study through the CCI, psychological impairment, and HRQoL in HD patients is complex and multidimensional. In the present study, higher CCI scores were associated with increased levels of depression, anxiety, and stress, as well as with lower HRQoL scores. These results are consistent with recently published data showing that a higher comorbidity burden amplifies psychological distress and decreases the subjective perception of well-being [59,60,61,62].

Regarding spKt/V, although dialysis adequacy is an essential foundation for survival, the literature provides less consistent correlations with mental disorders [63] and HRQoL [64], which is in agreement with our findings.

Available data in Romania on this subject remain limited regarding the complex evaluation of HRQoL in HD patients through instruments such as DASS-21R, correlated with clinical and paraclinical indicators such as CCI, spKt/V, and psychosocial factors.

From a clinical perspective, the weak correlations and small explained variance (R^2^ ≤ 0.15) observed in our models do not imply that psychological distress is unimportant, but rather that HRQoL in long-term HD is shaped by a wide range of determinants that extend beyond DASS-21R scores. Our cohort consisted of relatively stable, cognitively intact HD survivors with generally adequate dialysis (spKt/V mostly ≥ 1.2), which may have reduced the variability in some outcomes and attenuated statistical associations. In addition, several relevant domains were not directly measured in this study, including pain and pruritus severity, sleep disturbance, inflammatory activity, frailty, detailed social support quality, and coping strategies. Prior literature indicates that these factors can significantly influence HRQoL in CKD and HD populations, often interacting with mood symptoms rather than acting in isolation [65,66]. The small effect sizes and modest R^2^ are therefore consistent with a multifactorial, diffuse pattern in which psychological distress represents only one component of a broader biopsychosocial burden.

National-level studies confirm the increased prevalence and mortality of CKD [67,68], with a decrease in HRQoL for more than one-third of patients, under the influence of older age, female gender, longer dialysis duration, and low SES [69].

Recently, a series of interventions have been documented that can lead to an increase in HRQoL, an aspect that can only be achieved through integrated therapeutic approaches. Within this multidimensional framework, mental health emerges as a central domain that is closely intertwined with all other factors examined in our cohort, even though the cross-sectional design precludes establishing the direction of these relationships.

First of all, regular screening of mental health and HRQoL is necessary by introducing, in the routine evaluation of patients undergoing HD, standardized instruments such as DASS-21R and KDQOL-SF™ 1.3, for the early identification and management of vulnerable patients [53].

Multicentric studies have demonstrated a significant reduction in psychological symptoms and an increase in HRQoL following the implementation of psychological counseling programs, through short cognitive-behavioral therapies and relaxation techniques [70,71,72].

In parallel, educational–motivational interventions aimed at disease management, structured physical exercise, and nutrition can improve treatment adherence and increase the physical dimension of HRQoL [73,74,75].

Additionally, the implementation of social support programs involving family members and multidisciplinary teams has led to a reduction in psychological symptoms and an increase in HRQoL, with social improvements and reduced hospitalizations [6,76]. Nevertheless, new methods, like immersive virtual reality, could potentially improve mental health and wellbeing [77].

Descriptive data from the one-year reassessment support this interpretation. Among the 125 surviving patients, the overall distributions of DASS-21R severity categories and KDQOL-SF™ 1.3 domain scores were broadly similar to those observed at baseline, with persistent impairment in disease-specific burden and physical HRQoL and relatively preserved mental HRQoL. These findings are compatible with a relative short-term stability of psychological distress and HRQoL profiles in this stable, well-dialyzed survivor cohort. Nonetheless, the present analyses were not designed to formally model within-patient change, and more detailed longitudinal evaluations of trajectories and their determinants fall outside the scope of this cross-sectional report and will need to be addressed in future work. From a practical standpoint, our findings suggest that psychological distress screening should be viewed as a core, but not stand-alone, component of HD care pathways. Given the high prevalence of at least mild depressive, anxiety and stress symptoms, we support the routine use of a brief standardized instrument such as the DASS-21R in maintenance HD units, at the start of dialysis and then at regular intervals. However, the weak cross-sectional associations with PCS and MCS indicate that screening results should not be used in isolation to triage patients or to predict HRQoL outcomes. Instead, prioritization of interventions should focus on patients with moderate-to-extremely severe distress in combination with other markers of vulnerability (low SES, high comorbidity, severe disease-specific burden, poor physical functioning), and should ideally be delivered through multidisciplinary teams involving nephrologists, psychologists, psychiatrists, nurses and social workers. In this context, psychological care is best integrated into broader symptom-management and social support strategies rather than implemented as a narrow, stand-alone intervention.

## 5. Limitations

Our study presents a series of important limitations that should be mentioned. First, the sample size is relatively small and all patients were recruited from a single HD center, which limits the generalizability of the findings to the wider Romanian HD population. Second, the design is cross-sectional, with one main assessment and a descriptive one-year re-evaluation; therefore, we cannot infer temporal trajectories or causal relationships between mood symptoms, HRQoL and clinical parameters. Third, effect sizes for the associations between DASS-21R domains, clinical variables and HRQoL were generally small (with Spearman’s ρ mostly below 0.25 and R^2^ values ≤ 0.15 in multivariable models), underlining the exploratory nature of the analyses. In addition, because we examined multiple correlations and regression coefficients without applying a formal correction for multiplicity, the probability of type I error is increased and some nominally significant findings may be spurious. Consequently, the present regression models have limited utility for individual risk prediction, and our findings should be regarded as hypothesis-generating rather than as a basis for prognostic tools. Longer multicenter longitudinal studies with larger samples and repeated measurements would be needed to characterize the dynamic interplay between psychiatric symptoms, dialysis-related factors and HRQoL in more detail.

Several additional limitations should also be acknowledged. Psychological symptoms and HRQoL were assessed exclusively using self-reported questionnaires, which may be susceptible to reporting bias, social desirability effects, and transient emotional states at the time of assessment. Furthermore, the absence of structured clinical psychiatric interviews precludes the confirmation of formal psychiatric diagnoses and may have resulted in some degree of symptom misclassification. Residual confounding cannot be excluded, as relevant factors such as coping strategies, family and social support, treatment adherence, psychotropic medication use, and prior psychiatric history were not comprehensively assessed. Survivor bias may also be present, as only patients undergoing maintenance HD at the time of evaluation were included. Moreover, because we deliberately excluded patients with clinically relevant cognitive impairment (MMSE ≤ 20), those under ongoing psychotropic treatment for major psychiatric disorders, and individuals who died during the observation window, our analytic cohort likely represents a relatively psychologically and clinically healthier subset of the HD population; as a result, the prevalence and severity of psychological distress reported here are probably conservative estimates and may underestimate the true burden in more vulnerable patients. Finally, although a Romanian-language version of the KDQOL-SF™ 1.3 was employed, the lack of large-scale psychometric validation studies in Romanian HD populations should be considered when interpreting the HRQoL findings.

Multicenter studies with large samples and extensive follow-up periods, using standardized instruments that include complex clinical assessments and prompt interventions, are needed to confirm and extend these results.

## 6. Conclusions

Our study revealed a high prevalence of depression (48.0%), anxiety (34.4%) and stress (44.0%) among Romanian patients undergoing HD, together with marked impairment of physical HRQoL (PCS 36.49 ± 9.61) and comparatively less associated mental HRQoL (MCS 48.83 ± 8.76). In exploratory multivariable proportional-odds models, depression severity was independently associated with female sex and occupational score, while higher education, higher income and MMSE were protective; occupational score was also associated with anxiety severity, whereas no independent correlates were identified for stress severity. In line with previous reports, dialysis adequacy as assessed by spKt/V, while essential for prognosis, did not meaningfully account for the variability in HRQoL or DASS-21R scores.

The results emphasize the need for periodic screening, diagnosis, and monitoring of mental health, as well as for integrated, multidisciplinary, and individually tailored approaches that include psychosocial, educational, and physical support interventions aimed at optimizing HRQoL in HD patients in Romania. Such measures are expected to enhance treatment adherence, improve prognosis, and reduce mortality and morbidity.

We consider mental health to be the most important modifiable and rapidly adaptable dimension through integrated interventions. Thus, while our data support systematic psychological screening in HD units, they also indicate that changes to care pathways should emphasize integrated, multidisciplinary assessment and intervention for those with combined psychological, clinical and socioeconomic vulnerability, rather than relying on distress scores alone as a triage tool.

## Figures and Tables

**Table 1:** Categorical baseline sociodemographic and clinical characteristics (n = 125).

Variable	Category	Frequency (n (%))
Age class	<50 years	22 (17.60%)
	50–64 years	50 (40.00%)
	≥65 years	53 (42.40%)
Sex	Female	61 (48.80%)
	Male	64 (51.20%)
Residence	Rural	67 (53.60%)
	Urban	58 (46.40%)
Education	Primary school	5 (4.00%)
	Gymnasium	42 (33.60%)
	Vocational school	19 (15.20%)
	High school	38 (30.40%)
	Post-secondary	3 (2.40%)
	University	18 (14.40%)
SES category	Low (0–2)	43 (34.40%)
	Medium (3–4)	65 (52.00%)
	High (5–6)	17 (13.60%)
CCI category	1–2	11 (8.80%)
	3–4	33 (26.40%)
	≥5	81 (64.80%)
spKt/V category	<1.2	15 (12.00%)
	1.2–1.4	21 (16.80%)
	>1.4	89 (71.20)

**Table 2:** Distribution of DASS-21R depression, anxiety, and stress severity categories by sex and age class (n = 125).

Variable		n	Normal n (%)	Mild n (%)	Moderate n (%)	Severe n (%)	Extremely Severe n (%)
Depression							
Sex	Female	61	25 (40.98)	5 (8.20)	12 (19.67)	7 (11.48)	12 (19.67)
	Male	64	40 (62.50)	2 (3.12)	4 (6.25)	8 (12.50)	10 (15.62)
	*p*-value *	0.063					
Age class	<50 years	22	13 (59.09)	1 (4.55)	5 (22.73)	1 (4.55)	2 (9.09)
	50–64 years	50	23 (46.00)	2 (4.00)	8 (16.00)	5 (10.00)	12 (24.00)
	≥65 years	53	29 (54.72)	4 (7.55)	3 (5.66)	9 (16.98)	8 (15.09)
	*p*-value *	0.264					
Anxiety							
Sex	Female	61	39 (63.93)	11 (18.03)	7 (11.48)	4 (6.56)	0 (0.00)
	Male	64	43 (67.19)	2 (3.12)	11 (17.19)	6 (9.38)	2 (3.12)
	*p*-value *	0.047					
Age class	<50 years	22	17 (77.27)	2 (9.09)	1 (4.55)	0 (0.00)	2 (9.09)
	50–64 years	50	28 (56.00)	7 (14.00)	8 (16.00)	7 (14.00)	0 (0.00)
	≥65 years	53	37 (69.81)	4 (7.55)	9 (16.98)	3 (5.66)	0 (0.00)
	*p*-value *	**0.022**					
Stress							
Sex	Female	61	31 (50.82)	6 (9.84)	13 (21.31)	9 (14.75)	2 (3.28)
	Male	64	39 (60.94)	6 (9.38)	7 (10.94)	8 (12.50)	4 (6.25)
	*p*-value *	0.498					
Age class	<50 years	22	15 (68.18)	3 (13.64)	2 (9.09)	2 (9.09)	0 (0.00)
	50–64 years	50	25 (50.00)	4 (8.00)	12 (24.00)	7 (14.00)	2 (4.00)
	≥65 years	53	30 (56.60)	5 (9.43)	6 (11.32)	8 (15.09)	4 (7.55)
	*p*-value *	0.518					

* Chi-square test. Bold *p*-values indicate statistically significant associations at *p* < 0.05.

**Table 3:** KDQOL-SF™ 1.3 domain scores (n = 125).

Variable	Mean ± SD	95% CI
**Kidney disease-specific scales**		
Symptoms/problems list	85.13 ± 12.23	82.99–87.28
Effects of kidney disease	59.51 ± 18.34	56.30–62.73
Burden of kidney disease	43.90 ± 18.13	40.72–47.08
Work status	5.60 ± 17.06	2.61–8.59
Cognitive function	87.09 ± 12.26	84.94–89.24
Quality of social interaction	80.75 ± 13.79	78.33–83.16
Sexual function	55.40 ± 36.90	48.93–61.87
Sleep	70.44 ± 13.49	68.07–72.81
Social support	88.53 ± 21.52	84.76–92.31
Dialysis staff encouragement	85.80 ± 14.51	83.25–88.34
General health (KDQOL-SF^TM^ 1.3 global health rating)	62.40 ± 14.94	59.78–65.02
Patient satisfaction	79.20 ± 17.41	76.15–82.25
**SF-36–derived generic scales**		
Physical functioning	58.36 ± 23.75	54.20–62.52
Role-physical	43.40 ± 45.70	35.39–51.41
Pain	64.28 ± 21.90	60.44–68.12
General health (SF-36)	43.92 ± 12.98	41.65–46.19
Emotional well-being	70.30 ± 13.87	67.87–72.74
Role-emotional	69.60 ± 42.97	62.07–77.13
Social functioning	64.60 ± 23.59	60.47–68.73
Energy/fatigue	56.48 ± 15.88	53.70–59.26
PCS	36.49 ± 9.61	34.81–38.18
MCS	48.83 ± 8.76	47.29–50.36

All scores are expressed on a 0–100 scale; higher values indicate better perceived HRQoL.

**Table 4:** Spearman correlations (ρ) between DASS-21R scores and HRQoL and clinical variables (n = 125).

Variable	Depression ρ (*p*)	Anxiety ρ (*p*)	Stress ρ (*p*)
HRQoL domains			
Burden of kidney disease (KDQOL-SF^TM^ 1.3)	0.07 (0.417)	0.21 (0.018)	−0.09 (0.314)
KDQOL-SF^TM^ 1.3 global health rating	−0.06 (0.516)	0.19 (0.038)	0.06 (0.528)
General health (SF-36)	−0.08 (0.355)	0.19 (0.036)	0.07 (0.442)
Energy/fatigue	0.00 (0.987)	0.22 (0.015)	−0.03 (0.751)
PCS	−0.07 (0.435)	0.11 (0.222)	0.10 (0.290)
MCS	0.11 (0.206)	0.14 (0.117)	−0.12 (0.194)
Role-emotional (KDQOL-SF^TM^ 1.3)	0.12 (0.177)	0.04 (0.662)	−0.20 (0.024)
Clinical and socioeconomic variables			
SES score	−0.13 (0.140)	0.00 (0.973)	0.11 (0.209)
CCI	−0.03 (0.748)	0.08 (0.349)	0.16 (0.069)
spKt/V	0.01 (0.913)	−0.12 (0.186)	−0.02 (0.814)
Age	0.01 (0.871)	0.10 (0.291)	0.09 (0.314)

Values are Spearman’s ρ (*p*-value).

**Table 5:** Multivariable linear regression models for PCS and MCS (n = 125).

Predictor	PCS β (95% CI)	*p*	MCS β (95% CI)	*p*
Age 50–64 vs. <50 years	−3.46 (−8.91 to 1.98)	0.210	−0.72 (−5.53 to 4.09)	0.767
Age ≥65 vs. <50 years	−4.85 (−10.22 to 0.52)	0.076	0.12 (−4.63 to 4.87)	0.961
Male sex (vs. female)	1.29 (−2.39 to 4.96)	0.490	0.49 (−2.76 to 3.74)	0.766
SES score (per 1-point increase)	−0.33 (−1.71 to 1.05)	0.641	0.22 (−1.00 to 1.44)	0.719
CCI (per 1-point increase)	0.69 (−0.25 to 1.64)	0.149	−0.36 (−1.19 to 0.47)	0.395
spKt/V (per 1.0-unit increase)	2.90 (−2.43 to 8.23)	0.284	−2.79 (−7.50 to 1.93)	0.244
DASS-21R depression score	−0.87 (−1.96 to 0.23)	0.119	0.70 (−0.27 to 1.66)	0.155
DASS-21R anxiety score	1.73 (0.05 to 3.40)	0.043	1.68 (0.20 to 3.17)	0.026
DASS-21R stress score	−0.06 (−1.29 to 1.17)	0.924	−1.64 (−2.73 to −0.56)	0.003

Coefficients are unstandardized β (two decimals), with 95% confidence intervals; *p*-values are reported to three decimals. Model fit: PCS model R^2^ = 0.09 (adjusted R^2^ = 0.02; global *p* = 0.228); MCS model R^2^ = 0.15 (adjusted R^2^ = 0.08; global *p* = 0.027); DASS-21R depression, anxiety and stress correspond to standardized z-scores.

**Table 6:** Multivariable ordinal logistic regression models for DASS-21R severity categories at baseline (T1, n = 125).

Predictor	Depression aOR (95% CI)	*p*	Anxiety aOR (95% CI)	*p*	Stress aOR (95% CI)	*p*
Sex (female vs. male)	3.06 (1.34–7.00)	0.008	1.11 (0.47–2.62)	0.815	1.34 (0.60–2.99)	0.476
Residence (urban vs. rural)	1.17 (0.53–2.61)	0.697	1.34 (0.59–3.08)	0.487	1.90 (0.88–4.10)	0.103
Age (per 1-year increase)	0.98 (0.95–1.02)	0.343	1.00 (0.97–1.04)	0.876	1.02 (0.98–1.06)	0.275
Dialysis vintage (per 1-year)	1.08 (0.99–1.17)	0.068	1.07 (0.98–1.16)	0.124	1.00 (0.93–1.08)	0.946
Education score (per 1-point)	0.54 (0.31–0.95)	0.031	0.77 (0.42–1.39)	0.382	1.03 (0.61–1.76)	0.905
Occupation score (per 1-point)	2.75 (1.39–5.43)	0.004	2.06 (1.03–4.10)	0.04	1.27 (0.66–2.43)	0.476
Income score (per 1-point)	0.42 (0.19–0.91)	0.027	0.56 (0.24–1.32)	0.184	1.23 (0.57–2.65)	0.597
CCI (per 1-point)	0.98 (0.81–1.19)	0.826	1.18 (0.97–1.45)	0.101	1.06 (0.88–1.27)	0.543
spKt/V (per 0.1 increase)	0.99 (0.89–1.10)	0.849	0.95 (0.84–1.07)	0.364	1.03 (0.93–1.16)	0.545
MMSE (per 1-point)	0.70 (0.55–0.87)	0.002	1.09 (0.87–1.37)	0.444	0.98 (0.79–1.20)	0.822

## Data Availability

The data presented in this study are available from the corresponding author upon request. The data are not publicly available due to privacy restrictions.

## Abbreviations

The following abbreviations are used in this manuscript:

CCI	Charlson Comorbidity Index
CKD	Chronic Kidney Disease
DASS-21R	Depression, Anxiety and Stress Scale—21R
eGFR	Estimated Glomerular Filtration Rate
HD	Hemodialysis
HRQoL	Health-Related Quality of Life
KDIGO	Kidney Disease: Improving Global Outcomes
KDQOL-SF™ 1.3	Kidney Disease Quality of Life—Short Form, version 1.3
MCS	Mental component summary
MMSE	Mini-Mental State Examination
NICE	National Institute for Health and Care Excellence
PCS	Physical component summary
PD	Peritoneal Dialysis
QoL	Quality of Life
RRT	Renal Replacement Therapy
SES	Socioeconomic status
SES-3	Socioeconomic Scale (three-dimensional: Education, Occupation, Household Income)
SF-36	Short Form–36 Health Survey
spKt/V	Single-Pool Kt/V (Dialysis adequacy)
WHO	World Health Organization
WHOQOL-BREF	World Health Organization Quality of Life—Brief Version
